# Mesenteric cyst manifested as obesity, gastroesophageal reflux, urinary incontinence, and abdominal mass during pregnancy—Case report and literature review

**DOI:** 10.1016/j.ijscr.2022.107366

**Published:** 2022-06-29

**Authors:** Francisco Aguilar-Espinosa, Rodolfo Salcedo-Vargas, Hiram Alfonso Galván-Bizarro, Carlos Rubén Rodríguez-Ramos, Erika Diana Barba-Jaramillo

**Affiliations:** a General Surgery, General Hospital of Zone 21, Mexican Institute of Social Security, Tepatitlan de Morelos, Jalisco, Mexico; b Pathological Anatomy, Santa Fe Memorial Hospital, Tepatitlan de Morelos, Jalisco, Mexico; c Medical Student, General Hospital of Zone 21, Mexican Institute of Social Security, Tepatitlan de Morelos, Jalisco, Mexico; d Anesthesiology, General Hospital of Zone 21, Mexican Institute of Social Security, Tepatitlán de Morelos, Jalisco, Mexico

**Keywords:** MC, mesenteric cyst, LC, Losanoff's classification, GER, gastroesophageal reflux, GA, gestational age, Nd, normal delivery, Recurrence, Mesenteric cyst, Simple mesothelial cyst, Pregnancy, Puerperium, Case report

## Abstract

**Introduction and importance:**

The mesenteric cyst (MC) is a rare entity, a benign lesion that causes the growth of an abdominal mass and other clinical presentations. The presentation of MC during pregnancy is even less frequent.

**Case presentation:**

A 34-year-old Mexican woman presented with a mesenteric cyst treated with laparotomy aspiration during the 16th week of pregnancy; the pregnancy was resolved by cesarean section without problems. Nevertheless, 17 months later, the lesion recurred. New assessment and surgical treatment with complete excision are performed without evidence of further recurrence.

**Clinical discussion:**

This case is essential due to the low frequency of association between pregnancy and mesenteric cyst. Incomplete resection, aspiration, and marsupialization of the lesion carry a high risk of recurrence. Therefore, the opportune moment to perform a complete resection of the lesion and avoid complications should be evaluated during pregnancy.

**Conclusion:**

MC should be considered a differential diagnosis in cystic lesions during pregnancy. Imaging studies, complete surgical resection, histological evaluation, and follow-up are necessary for adequate treatment.

## Highlights

• Mesenteric cysts are rare lesions, and their presentation is more variable during pregnancy.
• An incomplete resection carries an essential risk of recurrence.
• The ideal treatment includes preoperative imaging, complete excision, histopathological evaluation, and follow-up.
• The mesenteric cyst could be complicated with acute abdomen, intestinal obstruction, and mesenteric ischemia.

## 1 Introduction

MC originates from the lymphatic tissue and mesothelium of the serous cavities [1]. The first description of intra-abdominal MC was in 1507, and to date, about 1000 cases have been described. They are rare injuries and have an incidence of between 1:100,000 and 1:350,000 hospitalizations [2], [3]. The etiology is unknown but may be caused by an ectopic proliferation of lymphatic vessels in the mesentery without communication to the lymphatic system or by a lack of fusion of the mesentery layers. Its formation includes various causes, such as congenital, traumatic malignant, and infectious [4], [5], [6]. They can develop anywhere in the gastrointestinal mesentery or omentum, from the duodenum to the rectum [5]. Losanoff's classification (LC) [7] helps assess the location, extension, and prognosis of MC:

A. Type 1: Pedunculated lesion; resection does not compromise intestinal irrigation.
B. Type 2: Sessile lesion at the edge of the mesentery, the resection compromises the irrigation, and intestinal resection may be necessary.
C. Type 3: Lesion extending to the retroperitoneum can affect structures such as the aorta or cava, making a complete resection impossible.
D. Type 4: Multicentric lesion, involvement of intra-abdominal organs and retroperitoneum.

The appearance of the fluid content of the cyst depends on the degree of lymphatic stasis, protein content, bleeding, or pus so that the fluid may be serous, bloody, or chyle. Tomography and magnetic resonance imaging are helpful for the relevant preoperative information on anatomical location, organs involved, size or dimensions, and content characteristics [7]. Drainage of the cyst is a suboptimal treatment due to its high recurrence and risk of infection, so surgical resection is the treatment of choice [8].

The association of MC with pregnancy or puerperium is even less frequent. Only a few cases are reported, and the last reported cases were from five years ago [4], [9]. Following the SCARE guidelines [10], we present a case of MC treated with laparotomy and drainage during pregnancy; however, the lesion recurred a short time later. The authors treated this case in a public hospital in Mexico. In addition, a review of the literature is carried out.

## 2 Ethics statement

Written informed consent was obtained from the patient and the husband to publish this case report and accompanying images.

## 3 Presentation of case

A 34-year-old Mexican housewife suffers from obesity due to excess calories (BMI 47 kg/m^2^), no smoker, two pregnancies, two cesarean sections, does not suffer from other diseases, no relevant family history, does not take any meds, no smoking, drugs, or alcohol, and has no allergies. She began her condition four years before her second pregnancy with progressive heartburn symptoms, gastroesophageal reflux (GER), and urinary incontinence; the patient associated these symptoms with her obesity problem until abdominal growth and abdominal mass were presented. For a better understanding of the case, the timeline of critical clinical events is given:

**Table:** 

2012	First uncomplicated cesarean section.
2015	Progressively begins symptoms of the case.
2019	A) During obstetric surveillance of the second pregnancy, an abdominal cystic lesion ≥20 cm is detected by ultrasound, making it challenging to monitor intrauterine growth. B) Due to the above, it was decided to perform a laparotomy at week 16 of gestation by another medical group, reporting drainage of 9 l of fluid from the cyst without resection for suspicion of retroperitoneum involvement. In addition, histological analysis of the fluid said chronic inflammation without malignancy. C) Improvement of symptoms during the rest of the pregnancy, a second cesarean section at week 40 without complications
2020	Seventeen months after drainage of the lesion, she started again with the same symptoms.
2021	A) January: hospitalization for five days due to pneumonia by Coronavirus SARS-COV2 (CO-RADS-5), improving and without sequelae. B) November: seeks new medical attention due to persistent symptoms and sensation of abdominal growth.

**Fig. 1:**
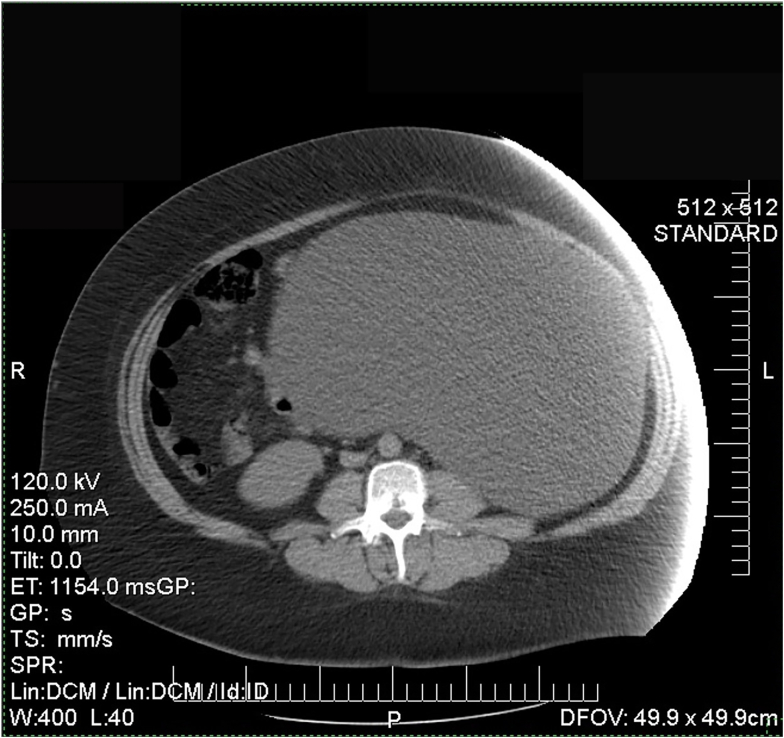
Simple and contrasted abdominal tomography.

The patient presented for review in the outpatient clinic, where preoperative and imaging studies were requested. On physical examination, the patient was found conscious, oriented, and showed vital signs: blood pressure 140/100 mm Hg, heart rate 74 beats per minute, respiratory rate 17 per minute, temperature 36.5 °C. In addition, on physical examination, an abdominal tumor was palpated from the hypochondrium, left flanks, mesogastrium, and an upbeat *Tillaux sing* (movement of the lesion horizontally). A contrast-enhanced abdominal tomography revealed a recurrence of a cystic lesion on the left flank measuring 25 × 16 × 28 cm, non-septated, without enhancing the contrast medium, showing displacement of bowel loops and uterine body (Fig. 1). Preoperative test and tumor markers did not alter (Table 1).

**Table 1:** Preoperative laboratory tests performed.

Test	Value	Test unit
*Complete blood count*		
Red blood corpuscles count	5.2	million/Al
Hemoglobin (Hb)	14.0	g/dl
Hematocrit (HCT)	44	%
Mean cell volume (MCV)	85.10	Fl
Mean cell hemoglobin (MCH)	27.10	Pg/cell
Mean cell hemoglobin concentration (MCHC)	31.80	%
Red cell distribution width (RDW)	14.20	%
Platelet count	220	thousand/Al
White cell count	7.3	thousand/mm^3^
Neutrophils	65 % (4.76)	%
Lymphocytes	6.0 % (1.96)	%
Monocytes	6.0 % (0.44)	%
Eosinophils	1.4 % (0.10)	%
Basophils	0.5 % (0.04)	%

*Coagulation profile*		
Prothrombin time (PT)	11.2	s
PT-INR	1.02	
Partial thromboplastin time (PTT)	33.9	s

*Clinical chemistry*		
Glucose	87	mg/dl
Urea	22	
BUN	10.28	
Creatinine	0.60	mg/dl
Cholesterol total	220	mg/dl

*Tumor markers*		
Ca-125	50	U/ml
Ca-15.3	7.3	U/ml
Carcinoembryonic antigen (CEA)	≤0.5	ng/ml

**Fig. 2:**
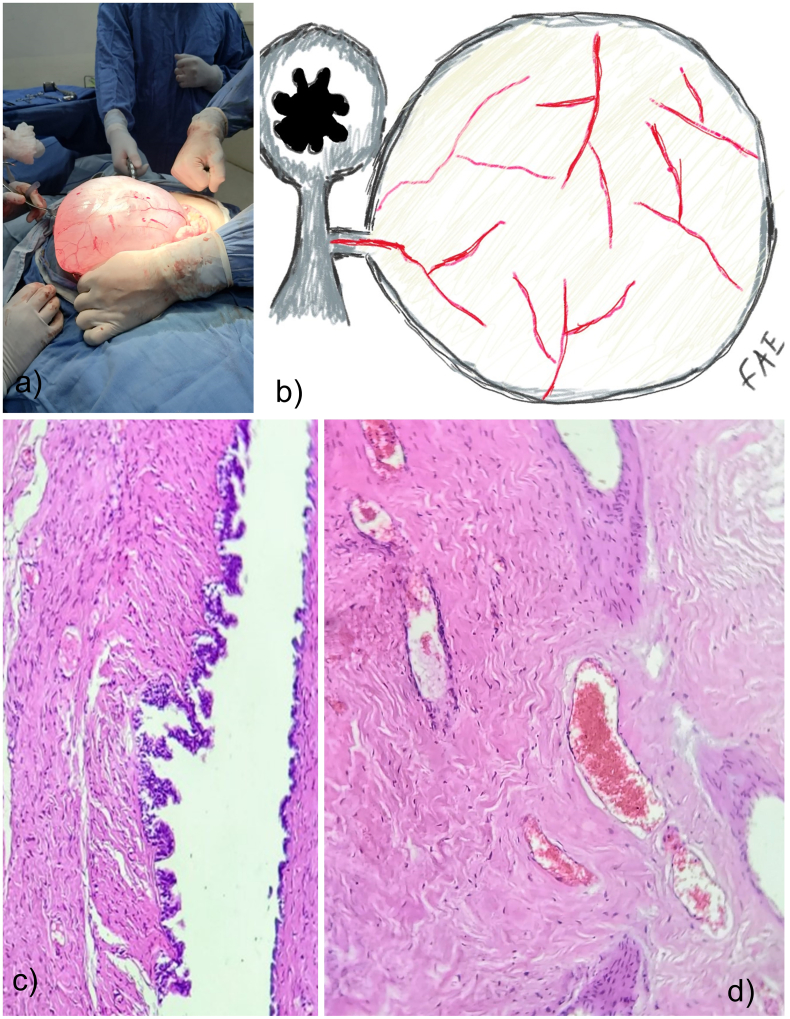
Intraoperative image shows large, unilocular, vascularized cystic lesion, liquid content, displacement of intestinal loops, and covering much of the peritoneal cavity (a). Diagram showing pedunculated lesion (LC Type 1) originating from the transverse mesocolon splenic angle (b). Histopathological examination of the excised MC revealed pseudostratified epithelium, which is continued by a hyalinized stroma with congestive vessels (c). Congestive vessels are surrounded by fibroconnective tissue; at higher magnification, these vessels are made up of flat endothelial cells and tapered fibroblasts with peripheral nuclei (d).

Due to the size of the recurrent lesion and previous cesarean sections that could cause adhesions, regional anesthesia laparotomy (midline incision) was decided in December 2021. The patient was prepared by fasting for 8 h, anti-embolic stockings 30 min before the procedure, Ceftriaxone 1 g, and Ondansetron 8 mg IV were used. During the surgery, the cystic lesion was found to be unilocular, covering a large part of the peritoneal cavity, causing displacement of intestinal loops and compression of the splenic flexure of the transverse colon against the retroperitoneum. Five liters of straw-colored fluid were drained, allowing resection of the lesion, using a Harmonic® scalpel (Harmonic Focus ± shears Ethicon Johnson-Johnson), adhered by loose adhesions and vascular pedicle from the mesentery of the splenic flexure of the transverse mesocolon (LC Type 1). The procedure was completed with concurrent appendectomy and the surgical time was 1.5 h, a minimum amount of blood loss. The first author performed the surgery, an attending physician, a certified general and bariatric surgeon with five years of experience, and a medical intern as the first assistant. The pathological analysis reports an appendix of 6 × 0.05 cm with appendicitis; ovoid cystic lesion 36 × 25 cm composed of a pseudostratified epithelium, capsule with fibroconnective tissue, lymphocytes, and plasma cells, and histocytes. The final diagnosis was a simple mesothelial cyst (Fig. 2).

During her post-surgical recovery, they used Ondansetron 8 mg iv every 12 h, Omeprazole 40 mg iv every 24 h, Metamizole 1 g iv every 8 h, Ketorolac 30 mg iv every 8 h, Ceftriaxone 1 g iv every 12 h and 60 mg of subcutaneous Enoxaparin. There were no postoperative complications, and the patient was discharged home with oral Cephalexin 500 mg every 8 h, oral Acetaminophen 500 mg every 8 h, and subcutaneous Enoxaparin 60 mg every 24 h for seven days. Four months later, continues the outpatient follow-up; the patient presents a resolution of symptoms and expresses complete satisfaction with the improvement in her quality of life and grants their consent to publish the present case, saying that it is helpful to resolve future similar issues. In addition, she had no evidence of recurrence of the lesion by control abdominal tomography.

## 4 Discussion

According to Perrot M [6], there are six types of mesenteric cysts due to their histopathological characteristics:

**Table:** 

1. Cysts of lymphatic origin A. Simple lymphatic cyst B. Lymphangioma
2. Cysts of mesothelial origin A. Simple mesothelial cyst B. Benign cystic mesothelioma C. Malignant cystic mesothelioma
3. Cysts of enteric origin A. Enteric duplication cyst B. Enteric cyst
4. Cysts of urogenital origin
5. Mature cystic teratoma (dermoid cyst)
6. Non-pancreatic pseudocysts A. Traumatic origin B. Infectious origin

Lymphangiomas are more frequent in children under 12, and mesothelial cysts are in young women [6]. The clinical presentation of MC can be in three ways: nonspecific abdominal symptoms, asymptomatic with an incidental finding on an imaging study or during surgery, and acute abdomen [4]. They are found more frequently in the mesentery of the ileum (60 %), ascending colon (24 %), and retroperitoneum (14.5 %) [8]. A retrospective case series (*n* = 16) showed that patients presented with abdominal pain (62 %), abdominal mass (43 %), and intestinal obstruction (6 %). The locations of the lesions were retroperitoneum (31 %), sigmoid mesocolon (25 %), and small intestine mesentery (25 %) [2].

Complete enucleation of the lesion by laparotomy or laparoscopy is the treatment of choice. Aspiration and marsupialization are associated with the risk of recurrence and infection. In the case of retroperitoneal involvement of large vessels, complete resection of the lesion is unlikely. The best option is partial resection with marsupialization into the peritoneal cavity [4], [8], [11]. 3 % of MC have malignant origin; lymphangiomas have a higher risk of recurrence (0 % to 27 % incomplete resection and 10 % to 100 % in incomplete resection) [7], [8]. When completely resected, cysts of mesothelial origin have a lower risk of recurrence (0–13.6 %) [8]. Postoperative clinical and radiological surveillance is recommended for three years [12].

The association between MC and pregnancy is rare, and only a few reported cases are reported. The mesenteric cyst is more common in women of childbearing age and can occur at any week of pregnancy or puerperium [4]. During pregnancy, it has been reported that lymphangiomas can increase in size due to the stimulation of uterine growth and an increase in cytokines, such as vascular endothelial growth factor [13]. Our review shows, including the present case, 17 cases of MC during pregnancy and puerperium have been reported from 1965 to date. Table 2 summarizes the clinical characteristics of the 17 cases. The most reported symptoms are distension and palpable abdominal mass (*n* = 12). Sessile cysts (LC Type 2) that affect the intestinal lumen and mesenteric irrigation cause intestinal obstruction and mesenteric thrombosis (*n* = 2). In other cases, it presented as ultrasound findings (*n* = 1), threatened abortion (*n* = 1), and epigastric pain with irradiation to the right hypochondrium (*n* = 1). Obesity and MC with a displacement of intra-abdominal structures can exacerbate GER and urinary incontinence due to increased intra-abdominal pressure [14], [15], as in the present case. Obesity was presented in 17 % of the cases of MC in pregnancy and puerperium.

**Table 2:** MC in pregnancy and puerperium.

Author and year	#	Age	GA diagnosis	Clinic	Surgery	Histology/LC	Complications	Days in hospital and outcome
Hill VL Jr, 1965 [17]	1	32	36	Abdominal distension	Cyst resection (6 weeks postpartum)	Simple mesothelial cyst/Type 3	None	Six days, nr
Dunn JM, 1967 [18]	2	22	18	Abdominal mass, vomiting, diarrhea, upper quadrant pain.	Cyst resection and intestinal resection 30 cm	Simple mesothelial cyst/Type 2 (mesentery of the ileum)	Transfusion (Hb 8 mg/dl), surgical wound infection	14 days; Nd at 40 gestation weeks
O'Driscoll, 1977 [19]	3	31	7	Abdominal mass	Cyst resection at six gestation weeks	Lymphangioma (16 × 20 cm)/Type 1 (mesentery of the ileum)	Transsurgical transfusion of 2 GP	Ten days; Nd
Rahatzad MT, 1986 [20]	4	19	11	Abdominal mass	Cyst resection	Enteric cyst (8 × 12 cm)/Type 3	None	Nr
Rahatzad MT, 1986 [20]	5	20	27	Abdominal growth is more significant than gestational age.	Cyst resection	Simple mesothelial cyst/Nr	None	Nr
Cohen I, 1988 [21]	6	36	Late puerperium	Abdominal mass	Cyst resection	Mucinous cystic adenoma (40 cm)/Type 1 (mesentery of the ileum)	None	Nr
Gast MJ, 1989 [22]	7	24	21	Abdominal mass	Cyst resection	Simple mesothelial cyst (21 cm)/Nr	None	Nr
Liew SC, 1994 [4]	8	23	One week postpartum	Six years previous marsupialization of mesenteric cyst. Lesion recurrence: Epigastric pain, anorexia, abdominal distension, abdominal mass.	Cyst resection	Lymphangioma (18 cm)/Type 2 (lesser omentum and transverse mesocolon)	None	Seven days, Nd
Cipriano L, 2000 [12]	9	34	14	Threatened miscarriage, suspected ovarian cyst.	Cyst resection	Lymphangioma (35 × 18 cm)/Type 1 (mesentery of the ileum)	None	Nr; Nd at 40 gestation weeks
Al-Mulhim AA, 2003 [23]	10	23	20	Abdominal mass	Laparoscopic cyst resection	Simple mesothelial cyst (17 × 12 × 10 cm)/Type 1	None	Three days; Nd
Torashima Y, 2003 [24]	11	31	25	Overweight (BMI 25 kg/m^2^), ultrasound findings four months before pregnancy, and intestinal obstruction	Cyst resection, intestinal resection (30 cm ileum) at 100 cm from ileocecal valve	Lymphangioma (15 × 10 cm)/Type 2 (mesentery of the ileum)	None	Nr; Nd at 40 gestation weeks
Konstantinidis K, 2005 [25]	12	31	15	Acute right abdominal pain, leukocytosis	Laparoscopic cyst resection, intestinal resection	Lymphangioma/Type 2 (mesentery of the ileum)	None	Three days; Nd
Sagili H, 2007 [26]	13	17	20	Asymptomatic, serial ultrasound findings during pregnancy (cyst 13 × 6 cm)	Cyst resection (6 weeks postpartum)	Simple mesothelial cyst (15 × 13 × 6 cm)/Type 3 (retroperitoneal behind ascending colon from right iliac fossa to liver)	None	Nr
Lambregts KWFM, 2014 [27]	14	33	19	Asymptomatic; serial ultrasound findings during a 19-week pregnancy reveal a cyst 15 × 12 × 6 cm; seven years later: Bloating and abdominal pain in the left quadrant.	Laparoscopic cyst resection (7 years after pregnancy)	Enteric cyst (30 × 15 × 20 cm)/Type 1 (mesentery of the ileum)	None	One day; follow-up two weeks later with no evidence of recurrence
Ozdemir O, 2017 [13]	15	21	26	Epigastric pain radiating to the right upper quadrant	Cyst resection during cesarean section at 39 gestation weeks	Lymphangioma (15 × 5 cm)/Type 1 (lesser sac and lesser gastric curvature)	None	One year follow-up without recurrence
Giannos A, 2017 [9]	16	27	10	Obesity (BMI 37 kg/m^2^), acute abdomen: rebound in the right iliac fossa, vomiting, tachycardia, low-grade fever, leukocytosis	Resection of two cysts, 60 cm resection of jejunal necrosis, appendectomy for necrosis	Two simple lymphatic cysts (9 cm and 4 cm)/Type 2 (jejunal mesentery)	Transfusion (hemoglobin 7 g/dl), pulmonary thromboembolism, celiac trunk thrombosis, mesenteric venous thrombosis, abortion	11 days, nr
Present case	17	34	16	Obesity (BMI 47 kg/m^2^), GERD, urinary incontinence, abdominal mass. Cyst drainage at week 16 of gestation. Lesion and symptoms recurrence 17 months later	Cyst resection, concomitant appendectomy	Simple mesothelial cyst (36 × 25 cm)/Type 1 (transverse mesocolon)	None	Two days, three-month follow-up without recurrence.

GA: gestational age, Nr: not reported, Nd: normal delivery.

The MC treated with marsupialization and drainage initially, including in our case, presented recurrence (*n* = 2). Complete resection of the lesion was reported in all cases: cyst resection (*n* = 13), cyst resection plus small intestine segment (*n* = 4), cyst resection plus small intestine segment plus concomitant appendectomy (*n* = 1) and cyst resection plus concomitant appendectomy (*n* = 1). The timing of resection was laparotomy during pregnancy (*n* = 8), laparotomy resection during the puerperium (*n* = 4), laparoscopic resection during pregnancy (*n* = 2), resection during cesarean section (*n* = 1), diagnosis during pregnancy and recurrence, resection by laparotomy (*n* = 2). According to the LC, type 1 (*n* = 8), type 2 (*n* = 4), type 3 (*n* = 3), type 4 (*n* = 0) and not reported (*n* = 2). MC locations in order of frequency were ileal mesentery (*n* = 8), retroperitoneum (*n* = 3), lesser omentum plus transverse mesentery (*n* = 1), jejunal mesentery (*n* = 1), transverse mesentery (*n* = 1) and not reported (*n* = 3). According to the histological types of Perrot M [6], as in the present case, the most frequent type was a simple mesothelial cyst (*n* = 7), lymphangioma (*n* = 6), enteric cyst (*n* = 2), simple cyst lymphatic (*n* = 1) and mucinous cystadenoma (n = 1). Mucinous cystadenomas usually originate from the ovaries; very few cases affect other organs such as the pancreas, liver, spleen, and mesentery [16]. Only one case presented severe complications, such as abortion, mesenteric thrombosis, and pulmonary thromboembolism. There were no more severe complications for the mother or the newborn in the rest of the cases. In addition, no case of recurrence after resection was reported, and the most extended postoperative follow-up was one year. Despite having few case reports for a rare pathology, we can observe and adds to the existing literature that MC presented predominantly as an abdominal mass or acute abdomen during pregnancy or puerperium. Surgical treatment during pregnancy does not present a greater risk for the mother and the newborn in most cases; complete resection is the best option because it does not carry a risk of recurrence.

## 5 Conclusion

MC should be included in intraabdominal cyst lesions during pregnancy as a differential diagnosis since they are more frequent in young women. Imaging studies, complete resection, histopathology evaluation, and follow-up are necessary for treatment. In cystic lesions >20 cm, during pregnancy, the timing of complete resection by laparoscopy or laparotomy should be assessed to avoid complications and rule out malignancy and recurrence. Type 3 or 4 MC has a high recurrence rate if they are not completely resected.

## Consent

Written informed consent was obtained from the patient and the husband to publish this case report and accompanying images. On request, a copy of the written permission is available for review by the Editor in Chief of this journal.

## Provenance and peer review

Not commissioned, externally peer reviewed.

## Ethical approval

Written informed consent was obtained from the patient and the husband to publish this case report and accompanying images.

Moreover, the ethical approval has been exempted by our institution, considering that the case was written using retrospective and anonymous data.

## Funding

This research received no specific grant from the public, commercial, or not-for-profit funding agencies.

## Guarantor

Francisco Aguilar-Espinosa.

## Research registration number

This is a case report, and it is not registered.

## CRediT authorship contribution statement

FAE: Data collection, interpretation, writing paper
RSV: Interpretation
HAGB: data collection
CRRR: data collection
EDBJ: Writing paper and final revision.

## Declaration of competing interest

All authors declare no conflict of interest.

## Appendix A Supplementary data

Supplementary material Image 1
